# Chronic urinary retention in eunuchs

**DOI:** 10.4103/0970-1591.33733

**Published:** 2007

**Authors:** Sujata Patwardhan, Ajit Sawant, M. Nagabhushana, Radheshyam Varma, Mohammed Ismail

**Affiliations:** Department of Urology, LTMMC and LTMMGH, Sion, Mumbai - 400 022, India

**Keywords:** Eunuchs, retention

## Abstract

Eunuchs seek medical attention only when extremely distressed by symptoms. No scientific publication has highlighted the medical problems of eunuchs in India till date, probably because of lack of access to this community and their reluctance in seeking medical help. We evaluated four eunuchs in the last three years with chronic retention of urine due to urethral stenosis, caused by an incorrect method of amputation of the penis and urethra. Though the management of the problem is simple, the article highlights the traditional method of castration and penectomy which is practiced in Indian eunuchs which leads to urethral stenosis.

## INTRODUCTION

The eunuchs in Mumbai (Nirvan group) undergo traditional methods of castration described here. It can potentially lead to many urological problems secondary to chronic urinary retention which in turn is due to penile urethral stenosis. We present our experience in treating and interacting with these four eunuchs who presented to us with chronic urinary retention. We are unaware of the other urological problems they have due to the traditional method of castration they undergo, as they are not willing to avail of any medical help.

Treating these patients did raise various questions leading us to reflect on our society, the medical systems practiced by various people, their beliefs and the complexity of human sexuality. It would take patience and perseverance to develop a rapport and deduce the reasons why these healthy males decide to become Nirvan Eunuchs.

## CASE REPORT

**Figure 1:**
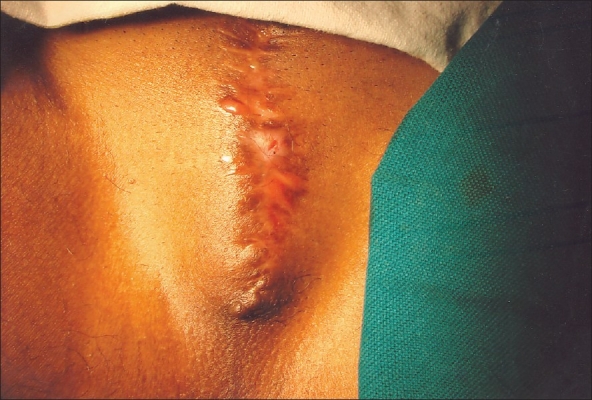
Picture showing stenosed urethra after amputation of penis

Four patients presented at the outpatient department with long-standing obstructive urinary symptoms and chronic painful retention. They needed 20 to 40 min to strain and empty the bladder in the squatting position. The age group was 27 to 34 years and they had become eunuchs by castration at the age of 18 to 20 years. They were garishly dressed as females with female mannerisms. All had obvious male secondary sexual features like close-shaven beard and moustache, male pattern of abdominal and pubic hair. Bladder was palpable and tender. Testis and penis were absent with a puckered scar at the suprapubic region and a stenosed external urinary meatus was seen a few centimeters below the symphysis pubis. The meatus could not be calibrated or dilated and was not clearly visible in one patient [Figure 1]. Routine biochemical investigations and ultrasound reports of the abdomen were within normal limits and one of the eunuchs was HIV positive. After an initial suprapubic cystostomy, an elective perineal urethrostomy was done. A request was made by two of the patients for creation of artificial vagina and vulva, but since it involved permission of their chief, they did not pursue the request later. None of the patients followed up one month after surgery.

## DISCUSSION

The eunuchs in Mumbai belong to two main groups: Aqua (who do not undergo penectomy and castration) and Nirvan (who undergo penectomy and castration).

The eunuchs do not disclose details about their past. Though they are polite, they expect preferential treatment and attention.

Views differ on the exact process of castration and one would believe that there are several procedures by which the Eunuchs (hijras) undergo castration and penectomy. We are aware of two practices described by our patients. A common practice, however, begins with the individual being sequestered in isolation for some days during which he is fed on a diet of opium and milk to keep him in a permanent state of intoxication. They worship the Goddess Durga in the form of Bahucharamata. On a day declared auspicious by the Guru, the boy is laid down on a hard surface and a cord is tied tightly around his testicles to stop the flow of blood. Several eunuchs hold him down as a sharp knife severs the penis and testicles in one swift movement. A metal or wooden plug is inserted into the wound to stop full closure and leave an aperture for the passage of urine. Hot oil is poured over the area and herbs are placed on it to hasten the healing process. The second method is to tie a thin but strong nylon thread around the penis and scrotum separately after the person is under the influence of alcohol. He is kept under the influence of alcohol for three days. The thread is tightened at regular intervals and kept in place till the scrotum and penis slough off. It usually takes two to three days. The wound is sutured by a local doctor or left to heal by use of turmeric powder and herbs. One of our patients also mentioned that nowadays the procedure is also done by qualified doctors under proper anesthesia in areas in Delhi. However, he was not willing to give further details. Some communities, however, do not consider the procedure complete until the boy has been made to sit on a grinding stone and pushed down until he bleeds from the anus. The drops of blood are taken to signify the first menstruation and only then is the initiation complete. Thereafter, the Guru takes over the proper upbringing of the newest member. The young hijra learns everything about the clan's customs and traditions at the feet of the Guru. His adopted family of fellow hijras provides a loving environment and he is fed, clothed and looked after well until he too feels a sense of security and well-being. We approached one of the local leaders to offer a survey of the entire group to detect and treat urethral problems but they were not interested in any survey or medical help. Monetary help was however welcome. Two of our patients expressed a desire to get artificial vagina to be reconstructed but did not pursue it. Only one reference article regarding Eunuchs in Chinese courts[1] mentions that with time the opening of the urethra could become narrowed despite use of dilators resulting in urinary dribbling or retention. In a documentary film by Gian Claudio Guiducci, it is mentioned that hundreds of men in the United States voluntarily choose to be castrated to reinvent their sexual identity for reasons other than sex reassignment. It is not clearly mentioned as to what their motivation is. Is the procedure legal? Should men have access to such an irreversible surgery on demand? Various sites on the internet mention the details of the procedure of castration in eunuchs which differ in different parts of the world, which are exhaustive and beyond the scope of a case report. We have highlighted the two methods described by our four patients. There could be more methods practiced elsewhere in India. The exact prevalence or incidence of urethral stenosis or other urological problems related to castration and penectomy in eunuchs in India is not mentioned in the literature.
